# Synthesis of manganese-based complex as cathode material for aqueous rechargeable batteries†

**DOI:** 10.1039/c8ra01982g

**Published:** 2018-04-26

**Authors:** Nan Qiu, Hong Chen, Zhaoming Yang, Sen Sun, Yuan Wang

**Affiliations:** Key Laboratory of Radiation Physics and Technology, Ministry of Education, Institute of Nuclear Science and Technology, Sichuan University Chengdu 610064 People's Republic of China qiun@scu.edu.cn wyuan@scu.edu.cn

## Abstract

A low-cost and eco-friendly system based on a manganese-based complex cathode and zinc anode was demonstrated. The cathode is able to reversibly (de-)insert Zn^2+^ ions, providing a high capacity of 248 mA h g^−1^ at 0.1 A g^−1^. *Ex situ* TEM and XRD were utilized to determine the electrochemical mechanism of this high capacity cathode. Moreover, the contribution of pre-added Mn^2+^ in electrolyte to the capacity was revealed, and nearly 18.9% of the capacity is ascribed to the contribution of pre-added Mn^2+^. With the help of additive, this aqueous rechargeable battery shows outstanding electrochemical property. Its cycling performance is good with 6% capacity loss after 2000 cycles at 4.0 A g^−1^, highlighting it as a promising system for aqueous rechargeable battery applications.

## Abstract 2

Manganese-based complex with high energy density shows a capacity retention of 94% over 2000 cycles.

## Graphical abstract

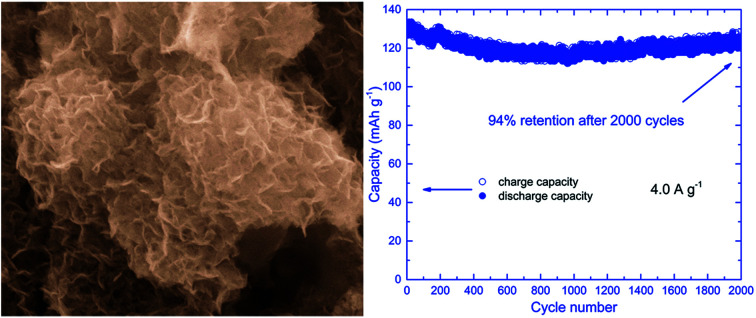

## 1. Introduction

Due to the ever-increasing environmental concerns and energy demands, eco-friendly and renewable energy technologies have attracted much attention.^1^ With the development of sustainable and clean energy sources such as tides, wind and solar, stable and efficient stationary energy storage systems (ESSs) are crucial for the utilization of those renewable energy sources.^2^ Among various alternatives, lithium-ion batteries (LIBs) have be considered as a leading candidate for mobile and digital devices.^3^ Although LIBs achieve high energy density,^4^ some intrinsic properties of LIBs hinder their large-scale applications in stationary ESSs, where the safety, cost and durability are the primary factors.^4^ Therefore, safe, low-cost and stable aqueous rechargeable batteries (ARBs) are the promising alternatives for large-scale stationary ESSs.

To date, a number of ARBs have been inspected, such as monovalent ions batteries,^5,6^ divalent ions batteries,^7,8^ trivalent aluminium-ion batteries and mixed-ion batteries.^9,10^ Development of aqueous zinc-ion batteries are particularly attractive and have attracted much interest.^4,11–13^ Recently, cation-defect enhanced ZnMn_2_O_4_ spinel has been demonstrated as a cathode material.^13^ Unfortunately, inferior capacity make this cathode less feasible for large-scale stationary ESSs. Additionally, cobalt oxide have been exploited as cathodes, which shows high reversible capacity, good rate performance and high stability.^14^ Nevertheless, the use of Co raise the cost and causes significant environmental concerns.^15^ On the other hand, manganese-based materials are relatively inexpensive because of the high abundance of manganese in the Earth's crust.^16^ Although manganese-based materials show some promise as cathodes for large-scale stationary ESSs, but the cathodes suffer poor rate-performance and significant capacity fading.^17,18^

In this work, we report an aqueous rechargeable battery based on Zn foil and low-cost manganese-based complex with high reversible capacity and long-cycle stability. The low energy consumption cathode exhibits an average discharge voltage of 1.32 V at 0.1 A g^−1^ and can be cycled at 100% coulombic efficiency at both low (0.1 A g^−1^) and high (4.0 A g^−1^) current density.

## 2. Experimental

### 2.1. Material synthesis

All chemical reagents were purchased from Aladdin Reagent Company (Shanghai, China) and used without any further purification. The synthetic procedure was conducted as follows: first, 2.535 g of MnSO_4_·H_2_O and 3.422 g of (NH_4_)_2_S_2_O_8_ was dissolved in 100 ml of deionized water (denoted as solution A). 2.16 g of NaOH was dissolved in 100 ml of deionized water (denoted as solution B). Then, solution B was dropwise added to solution A under vigorous stirring. The mixed solution was kept stirring for 1 hour and aged overnight at room temperature. The precipitate was collected by filtration, washed with water and ethanol, and subsequently dried at 50 °C.

### 2.2. Characterizations

X-ray diffraction (XRD) patterns of the samples were recorded on an Empyrean X-ray diffractometer using filtered Cu Kα radiation at 40 kV and 40 mA. The morphologies of the samples were characterized by SEM using a field emission scanning electron microscope (FE-SEM, JEOL, JSM-7500F). Transmission electron microscope (TEM) images were obtained using a ZEISS LIBRA-200FE field emission transmission electron microscope operating at 200 kV. X-ray photoelectron spectroscopy (XPS) experiments were performed in a Kratos XSAM 800 instrument.

### 2.3. Electrochemical measurements

The electrochemical tests were carried out in coin-type cells (CR2032). The working electrodes were prepared as follows: the as-prepared material was mixed with carbon black (Super P) and polymer binder (polytetrafluoroethylene, PTFE) in a weight ratio of 70 : 20 : 10 with the help of ethanol. After drying, the mixture was pressed into a film and cut into disk (diameter 10 mm, thickness ∼90 μm, see Fig. S1 in ESI†). The mass of cathode was measured by an electronic balance (Sartorius BSA124S, 0.1 mg resolution). Several cathode were weighed and then used. The manganese-based complex mass loading is 2–3 mg cm^−2^. The coin cells were assembled in open air atmosphere using a manganese-based complex electrode as the cathode, a glass fibre (GF/D, Whatman) as the separator and a zinc foil (Alfa Aesar) as the anode. 0.2 ml of 1 M ZnSO_4_ with 0.1 M MnSO_4_ as an additive in H_2_O was used as the electrolyte without oxygen removal.^19^ The charge–discharge (1.0–1.85 V *vs.* Zn/Zn^2+^) tests were conducted on a LANHE battery tester (Wuhan, China) at room temperature. The cells were cycled at various current densities. A two-step charge process is employed. That is, the constant current charge step is followed by an additional constant voltage charge step till the current drops to two fifths of its initial value. The cyclic voltammetry (CV) tests were carried out using a CorrTest (CS150) electrochemical work station.

## 3. Results and discussion

**Fig. 1:**
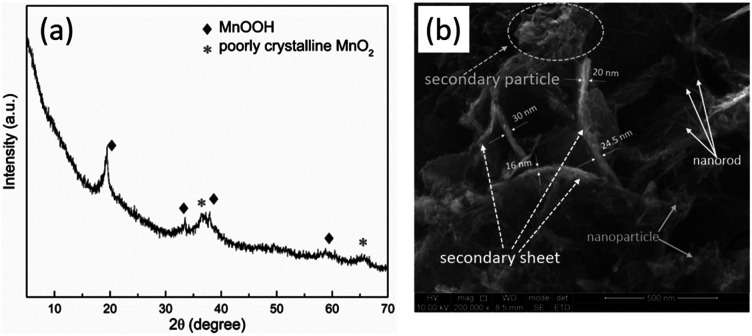
Structural and morphological characterization of as-prepared sample. (a) XRD pattern and (b) SEM image of the as-prepared manganese-based complex.

The phase purity and crystal structure of the synthesized products were examined by using XRD. As shown in Fig. 1, the mainly peaks in the XRD patterns of as-prepared are identified using the standard MnOOH database (JCPDS no. 18-0804).^20^ The peaks are observed at 36.6° and 65.3° (2*θ*) could be indexed in partially crystalline MnO_2_ (JCPDS no. 42-1169).^21,22^ The morphology of as-prepared sample was confirmed by using a field emission scanning electron microscope (FE-SEM). As shown in Fig. 1b, the as-prepared particles consist of nanoparticle and nanorod, which agglomerated to each other, forming secondary particles with the size of a few hundred nanometers, and secondary sheets of about 16–30 nm in thickness.

The electrochemical properties of facile synthesized manganese-based complex (MnOOH and MnO_2_) in aqueous rechargeable ion batteries was examined using coin-type (CR2032) cells. Based on the previous result, MnSO_4_ was used as additive to improve the cycling performance of manganese-based electrodes.^7^

**Fig. 2:**
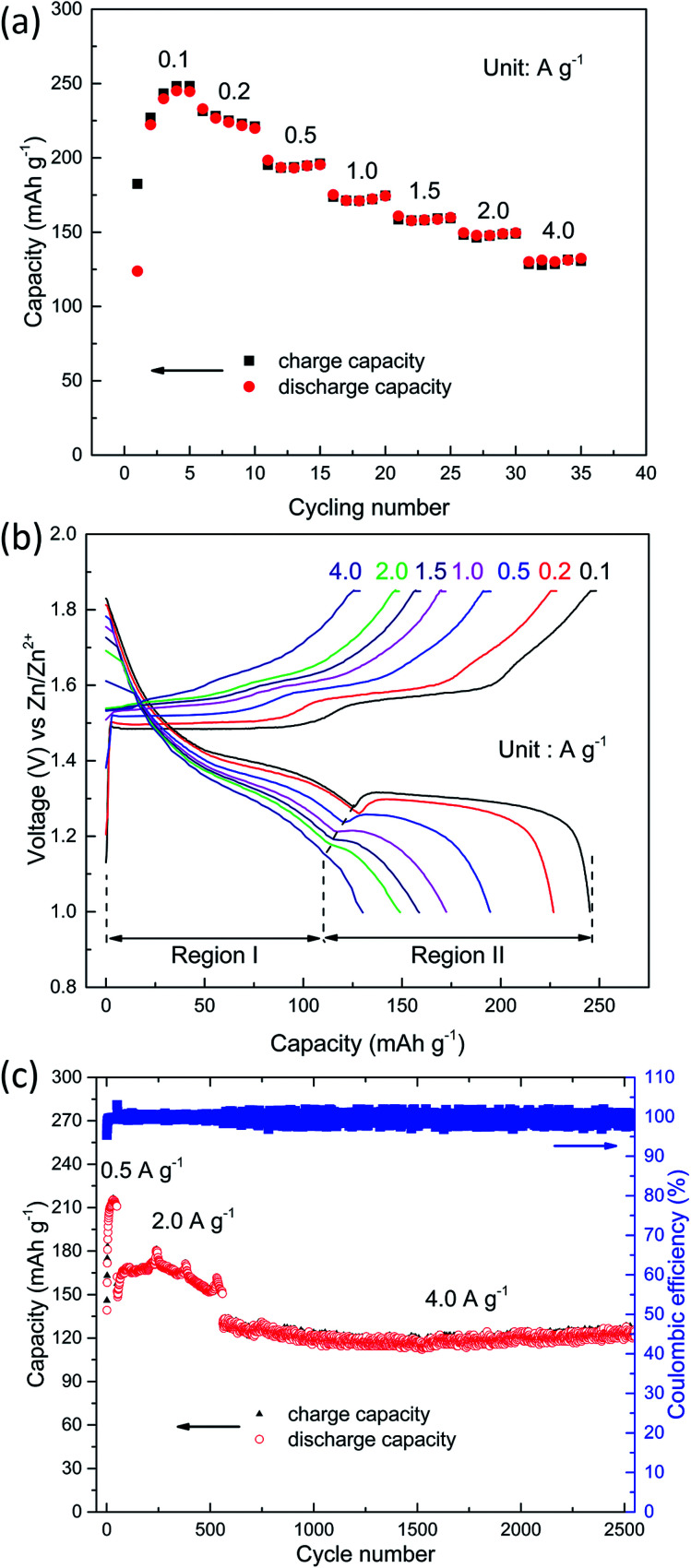
Electrochemical performance of manganese-based complex. (a) Cycling performance at various current densities (0.1–4.0 A g^−1^). (b) Charge and discharge voltage profiles at various current densities between 1.0–1.85 V *vs.* Zn/Zn^2+^. (c) Long-term cycling performance of manganese-based complex.

In addition to the rate capability test, the cells were cycled at various charge/discharge current rates ranging from 0.1 to 4.0 A g^−1^ (based on the mass of manganese-based complex) over a potential window of 1.0–1.85 V *vs.* Zn/Zn^2+^. The manganese-based complex electrode shows an excellent rate capability (see Fig. 2a), delivering high specific charge and discharge capacities of 248, 231, 196, 175, 160, 149 and 131 mA h g^−1^ at 0.1, 0.2, 0.5, 1.0, 1.5, 2.0 and 4.0 A g^−1^, respectively.

The charge and discharge curves are shown in Fig. 2b. A constant voltage charge step was added till the charge current dropped to two fifths of its initial value. A small fraction (less than 2.4%) of the total charge capacity was corresponding to the constant voltage step. Interestingly, with the increase in current density, the capacity drops in the first voltage plateau (denoted as region I, see Fig. 2b) are small, whereas capacity in the second voltage plateau (denoted as region II, see Fig. 2b) dramatically dropped, demonstrating that the reaction kinetics in the high plateau is extremely faster than the reaction in the low plateau.^23^ This is consistent with the results of CV analysis (see below). The average discharge voltage of this low-cost and safe ARB is about 1.32 V. The cathode delivers an average specific energy density of 335 Wh kg^−1^ at 0.1 A g^−1^. Even at high current density, the cathode still exhibits a superior energy density (Table S1, see ESI†). For example, the energy density is 198 Wh kg^−1^ at 2.0 A g^−1^. This is much higher than the values reported for aqueous Zn-ion batteries,^24–30^ as well as aqueous Li-ion batteries,^6,31^ or aqueous Na-ion batteries (Tables S1 and S2, see ESI†).^32,33^

For the evaluation of long-cycle stability, the cycling performance of manganese-based complex at 0.5, 2.0 and 4.0 A g^−1^ are shown in Fig. 2c. The manganese-based complex exhibited excellent cycling stability during repeated charge/discharge cycles. For the continuous cycling test, considerable capacity of 211 mA h g^−1^ is attained at 0.5 A g^−1^ after 50 cycles. The capacity retention for the manganese-based complex at 2.0 A g^−1^ is nearly 100% after 500 cycles, and high capacity of 150 mA h g^−1^ is obtained with high coulombic efficiency (∼100%). For the higher current density test, it delivers a reversible capacity of 124 mA h g^−1^ at 4.0 A g^−1^ after 2000 cycles, corresponding to 93.9% of its initial capacity.

**Fig. 3:**
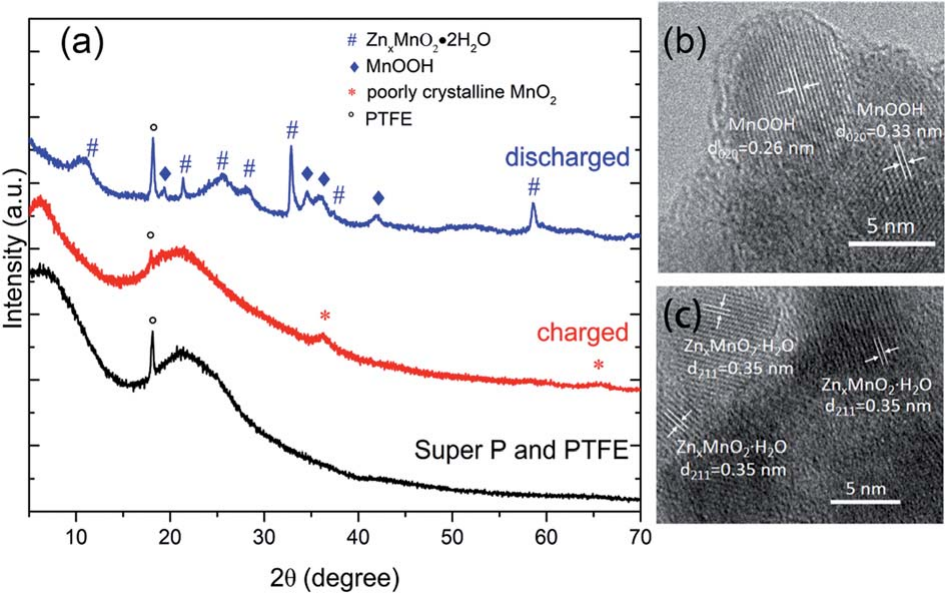
Electrochemical mechanism studies on manganese-based complex during charge and discharge process. (a) XRD patterns of binder and conductive agent, manganese-based complex electrodes in charged and discharged states. (b and c) HRTEM images of manganese-based complex electrodes discharged to 1 V.

**Fig. 4:**
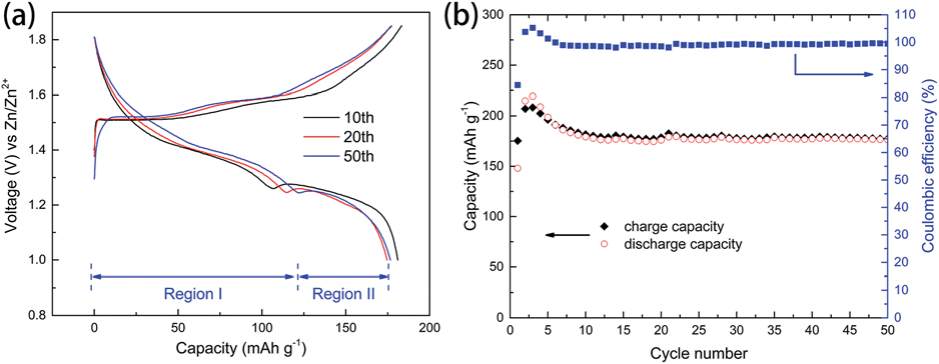
Electrochemical performance of manganese-based complex in the ammonia-adjusted electrolyte (pH = 6.0). (a) Charge and discharge voltage profiles at current density of 0.1 A g^−1^ in nearly neutral electrolyte (b) Cycling performance of manganese-based complex in nearly neutral electrolyte.

As shown in Fig. 3, *ex situ* XRD and TEM were used to understand the electrochemical mechanism of cathode. XRD pattern of charged electrode can be indexed to a cubic unit cell of the poorly crystalline MnO_2_ (JCPDS: 42-1169).^21,22^ For the discharged state, XRD pattern can be indexed to MnOOH (JCPDS: 74-1049)^7^ and Zn_*x*_MnO_2_·H_2_O (JCPDS: 47-1825).^34^ HR-TEM results (Fig. 3b and c) of discharged electrode confirmed the formation of MnOOH and Zn_*x*_MnO_2_·H_2_O. In the previous literature, the formation of MnOOH is attributed to the H^+^ insertion/extraction during discharge/charge processes.^23^ However, the concentration of H^+^ ions in the electrolyte (pH = 4.3) is very low, and it may not the reason for the excellent electrochemical performance. Besides, MnO_2_ electrode shows good stability in the neutral electrolyte.^35^ To reduce the effect of H^+^ ions, the pH value of the electrolyte was adjusted to 6.0 by adding ammonia. As shown in the Fig. 4, charge and discharge voltage profiles also show two plateaus in nearly neutral electrolyte. The results suggest that two plateaus may arise from the different sites in the MnO_2_. Moreover, hydrated zinc ion react with MnO_2_, and the accompanied water may provide OH^−^, which can produce MnOOH.

**Fig. 5:**
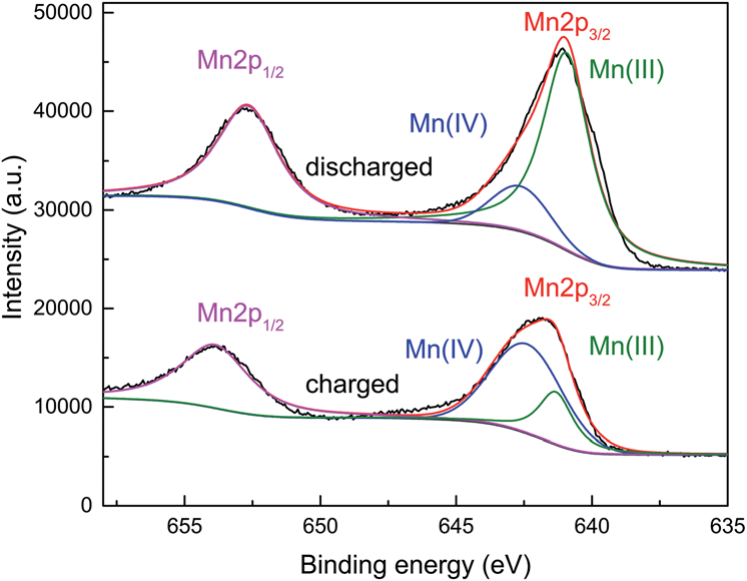
XPS spectra of the manganese-based complex electrodes in charged and discharged states.

The analysis of the oxidation states of the electrodes in charged and discharged states were carried out by XPS. As shown in the Fig. 5, Mn(iii) and Mn(iv) are the dominant in the discharged and charged state, respectively. It suggested that the capacity of this manganese-based complex is ascribed to the reversible reaction of Mn(iv)/Mn(iii).

Although the room-temperature synthesized manganese-based complex cathode with pre-addition of Mn^2+^ in electrolyte has been demonstrated excellent electrochemical performance, the underneath mechanism remains incompletely understood.

**Fig. 6:**
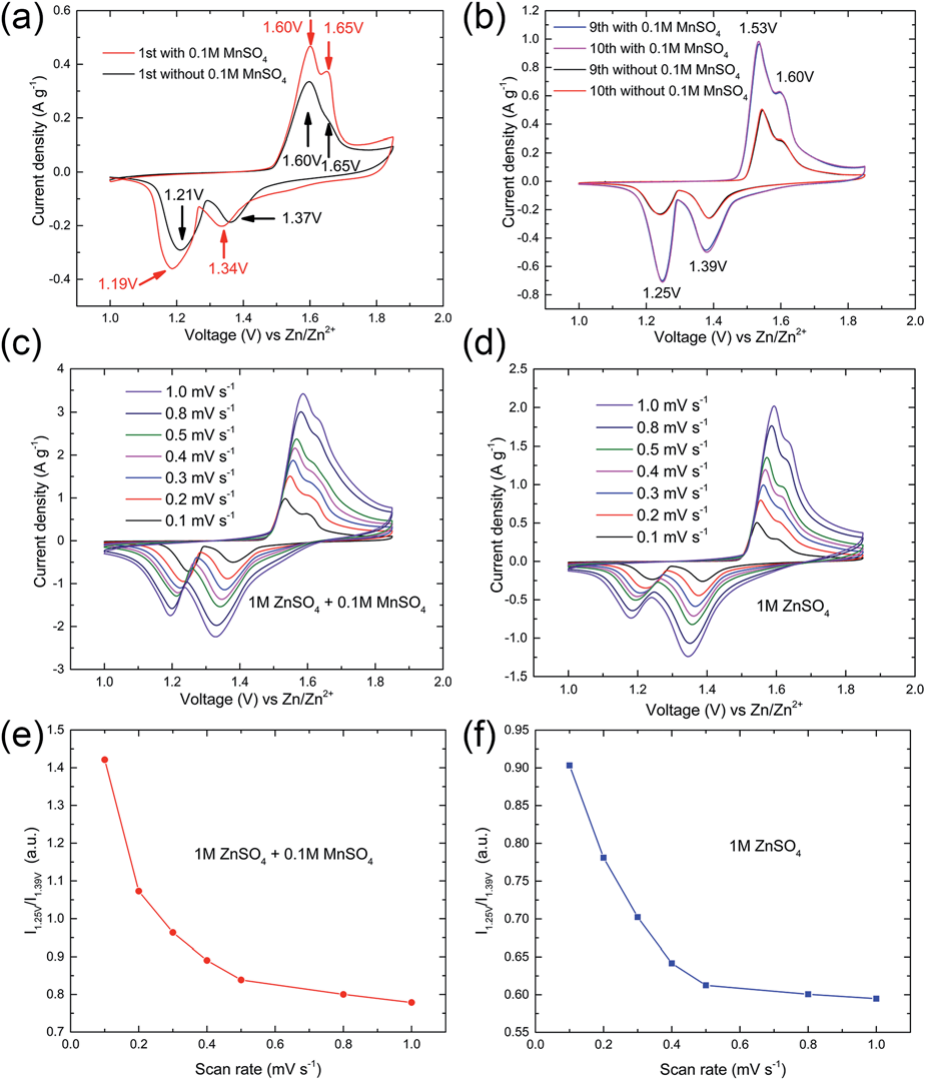
(a and b) CV curves of manganese-based complex electrode with/without 0.1 M MnSO4 scanning at 0.1 mV s^−1^. (c and d) CV curves of manganese-based complex electrode at different scan rates from 0.1 to 1 mV s^−1^. (e and f) The relative intensity *I*_1.25 V_/*I*_1.39 V_ gradually decay, *I*_1.25 V_ and *I*_1.39 V_ is intensity of peak located around 1.25 V and 1.39 V, respectively.

To further understand the function of Mn^2+^ electrolyte additive and the kinetics of the manganese-based complex electrode in this ARB, CV measurements were performed at various scan rates from 0.1 to 1.0 mV s^−1^. As shown in Fig. 6a, two separated reversible redox peaks can be clearly identified in the first cycle at 0.1 mV s^−1^. The position of redox peaks was slightly affected by the additive. Whereas, the current density was obviously increased, which indicated the pre-added Mn^2+^ ions play an important role in the subsequent charge/discharge processes. For both cells, there are two pairs reduction/oxidation peaks located at 1.25/1.53 and 1.39/1.60 V after the initial cycle (Fig. 6b), which implies pre-added Mn^2+^ ions did not affect the reaction mechanism for the manganese-based complex cathode in this ARB, but provide additional capacity (Fig. S3 in ESI†).

Two pairs reduction/oxidation peaks are in accordance with plateaus of the discharge/charge curves, corresponding to a two-step reaction. Besides, the CVs of manganese-based complex cathode remain invariable after several initial cycles, demonstrating the good reversibility. As presented in Fig. 6c–f, with the increase in CV scanning rate, the relative intensity *I*_1.25 V_/*I*_1.39 V_ gradually decay, where *I*_1.25 V_ and *I*_1.39 V_ is intensity of peak located around 1.25 V and 1.39 V, respectively. The results suggest that, as the current increases, the capacity fading rate at the upper voltage plateau (corresponding to region I) is slower than that at the lower voltage plateau (corresponding to region II), which is consistent with the results of the charge/discharge experiments. It also means that the kinetics of manganese-based complex electrodes is not influenced by pre-addition of Mn^2+^ in electrolyte.

**Fig. 7:**
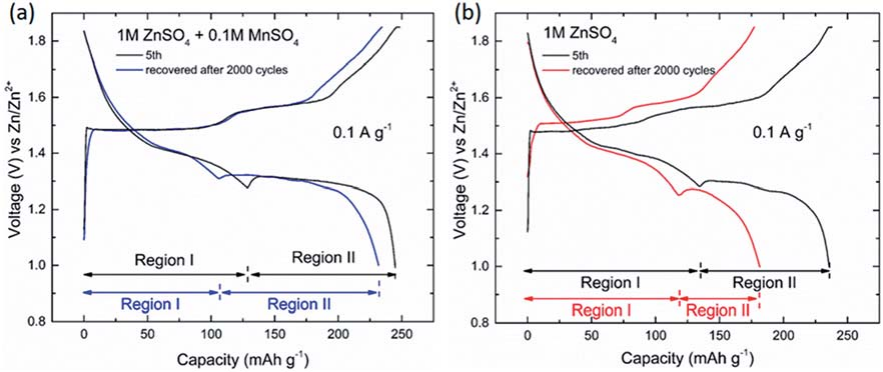
Charge/discharge voltage profiles of manganese-based complex cathode in the initial stage (5th) and recovered stage (after 2000 cycles at 4.0 A g^−1^) at low current density.

As for the long-cycle stability induced by the pre-addition of Mn^2+^, the cells were experienced low rate tests (5 cycles at 0.1 A g^−1^), subsequently long-term cyclic test (2000 cycles at 4.0 A g^−1^), and finally recovered at 0.1 A g^−1^. The results can be seen in Fig. 7, which clearly show that capacity of the region II was enhanced by the pre-addition of Mn^2+^ in electrolyte. The dissolution-inhibiting effect of Mn^2+^ form the manganese-based cathode may explain why the cell with MnSO_4_ additive shows high specific capacity. But why is Zn^2+^ insertion in the region II only severely affected, which is still unknown, unless the mechanisms involved in system are fully understood.

**Fig. 8:**
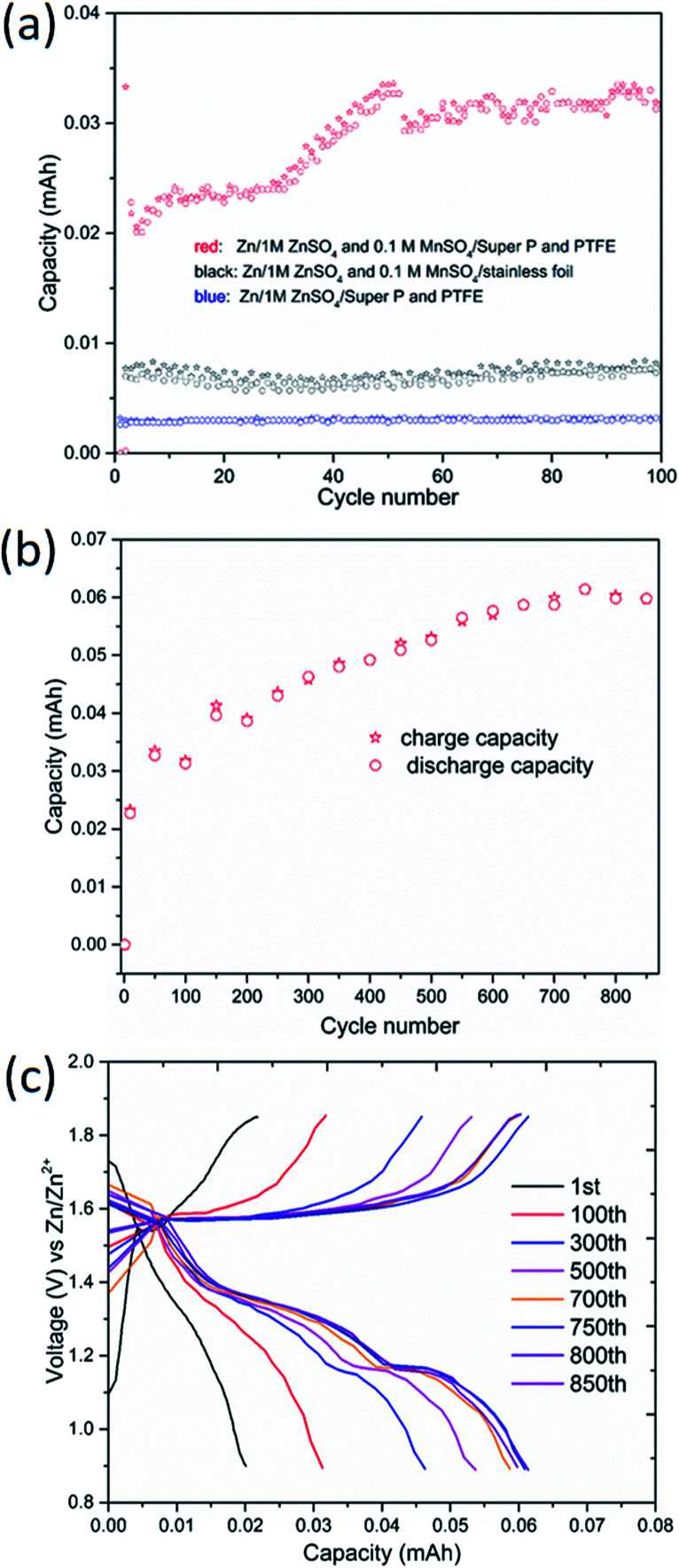
The effect of Mn^2+^ in electrolyte in this ARB. (a) Charge/discharge capacity of various cells at 2 mA cm^−2^ using 1 M ZnSO_4_ with/without 0.1 M MnSO_4_. (b) Long-term cycling performance of Zn/1 M ZnSO_4_ + 0.1 M MnSO_4_/Super P cell. The MnSO_4_ additive-generated manganese oxide could deliver a capacity of 0.059 mA h, which is nearly 18.9% for the reversible capacity (1.89 mg × 165 mA h g^−1^ = 0.312 mA h) of the active material. (c) Charge/discharge profiles of Zn/1 M ZnSO_4_ + 0.1 M MnSO_4_/Super P cell.

To probe the contribution of the extra capacity caused by Mn^2+^ electrolyte additive in this ARB, coin cells using different cathode electrodes were assembled. Homemade carbon current collector (Super P : PTFE = 9 : 1) and stainless foil served as cathode electrode, respectively. As shown in Fig. 8a, cell with conductive carbon delivered negligible capacity in the absence of Mn^2+^ in the electrolyte. However, the situation was different when the electrolyte containing 0.1 M MnSO_4_. The reversible capacity increased slowly every cycle in the initial stage, and continuously increased to 0.059 mA h, which is nearly 18.9% for the reversible capacity of the active material (see caption in Fig. 8). It is much higher than the one in the previous literature,^19^ and indicates that the contribution of pre-added Mn^2+^ in electrolyte to the cathode capacity cannot be neglected. Moreover, stable and less capacity can be obtained when the conductive carbon is substituted by stainless foil. The results suggest that the both conductive carbon and Mn^2+^ in the electrolyte are critical for the extra considerable capacity.

## 4. Conclusions

In summary, an aqueous rechargeable battery based on manganese-based complex and zinc foil using mild 1 M ZnSO_4_ electrolyte with/without 0.1 M MnSO_4_ was demonstrated. This low-cost and eco-friendly cathode delivers a capacity of 248 mA h g^−1^, an energy density of 335 Wh kg^−1^ at 0.1 A g^−1^. It shows a capacity retention of 100%, 93.9% over 500, 2000 cycles at 2.0, 4.0 A g^−1^, respectively. Poorly crystalline MnO_2_ is formed in the charged state, and it is able to reversibly insert Zn^2+^ ions. The effect of Mn^2+^ in electrolyte in this ARB also has been investigated. Nearly 18.9% of the capacity is ascribed to the contribution of pre-added Mn^2+^. This study could lead to a promising safe, low-cost and highly stable battery system for application in large-scale storage.

## Conflicts of interest

There are no conflicts to declare.

## Supplementary Material

RA-008-C8RA01982G-s001
