# Supplementary material for: Synthesis of manganese-based complex as cathode material for aqueous rechargeable batteries
Source: RSC Adv. 2018 Apr 26;8(28):15703–8. doi: 10.1039/c8ra01982g (PMC9080094; doi:10.1039/c8ra01982g)
Supplement: RA-008-C8RA01982G-s001 [file RA-008-C8RA01982G-s001.pdf]

Electronic Supplementary Material (ESI) for RSC Advances.
This journal is © The Royal Society of Chemistry 2018

Electronic Supplementary Information

Facile synthesis of manganese-based complex as cathode materials for conductive-carbon-assisted aqueous rechargeable batteries

Nan Qiu,* Hong Chen, Zhaoming Yang, Sen Sun, Yuan Wang*

Key Laboratory of Radiation Physics and Technology, Ministry of Education, Institute of Nuclear Science and Technology, Sichuan University, Chengdu 610064, People's Republic of China.
E-mail: qiun@scu.edu.cn, wyuan@scu.edu.cn.

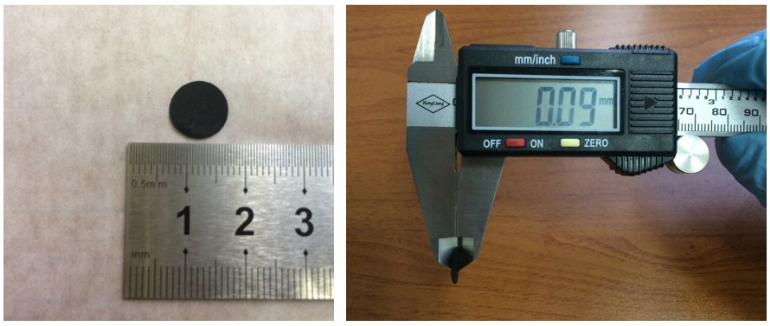

Figure S1. The photographs of the cathode film.

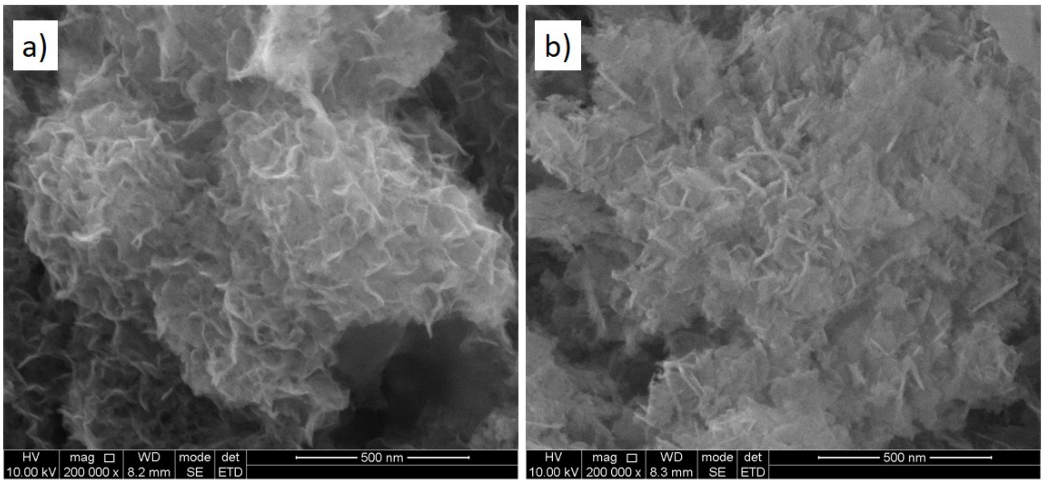

Figure S2. SEM images of cathode electrode in the charged/discharged state. a) 1.85V, charged state, b) 1V discharged state.

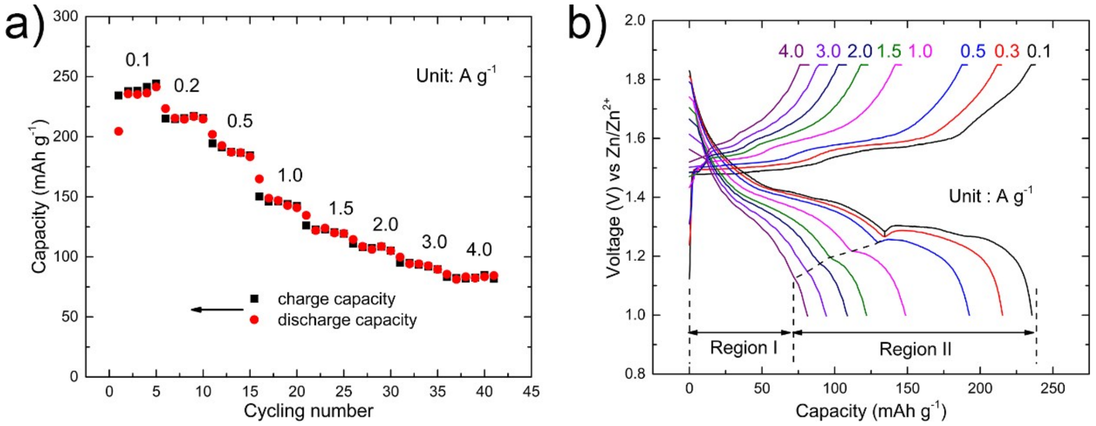

Figure S3. Electrochemical performance of manganese-based complex in 1M $ZnSO_4$. a) Cycling performance at various current densities (0.1-4.0 A g^{-1}). b) Charge and discharge voltage profiles using 1M $ZnSO_4$ as electrolyte at various current densities between 1.0-1.85 V vs. Zn/Zn^{2+}

Table S1. Specific capacity and energy density of manganese-based complex.

Current density ($A\ g^{-1}$)	0.1	0.2	0.5	1.0	1.5	2.0	4.0
Discharge capacity ($mAh\ g^{-1}$)	248	231	196	175	160	149	131
Energy density ($Wh\ kg^{-1}$)	335.0	317.3	261.5	231.0	211.8	197.5	175.0

Table S2. Summary of electrochemical performance of different cathode materials for aqueous rechargeable batteries

Samples	Electrolyte	Energy density base on the active mass of electrode materials	Cycling performance	Reference number
$LiMn_2O_4$	21M LiTFSI	~200 Wh/kg at 24 mA/g	~40 mAh/g<br>68% with 1000 cycles at 540 mA/g and 78% with 100 cycles at 18 mA/g	3
$LiMn_2O_4$	0.5 M Li_2SO_4	~75 Wh/kg at 500 mA/g	~120 mAh/g<br>100% capacity after 1200 cycles at 500 mA/g	4
$LiMn_2O_4$	0.5 M Li_2SO_4	~100 Wh/kg at 500 mA/g	37 mAh/g<br>93% capacity retained after 10000 cycles at 1000 mA/g	5
$NaMnO_2$	2 M CH3COONa	30Wh/kg at 60 mA/g	37 mAh/g<br>75% capacity retained after 500 cycles at 300	6
$Na_{0.95}MnO_2$	0.5M $Zn(CH_3COO)_2$<br>0.5M CH_3COONa	~84 Wh/kg at 1C	40 mAh/g<br>92% capacity retained after 1000 cycles at 4C	7
Amorphous $FePO_4$	1M $ZnSO_4$	--	96mAh/g at 10 mA/g	8
$ZnMn_2O_4$	3M $Zn(CF_3SO_3)_2$	~202 Wh/kg at 50 mA/g	~90 mAh/g<br>94% capacity retained after 500 cycles at 500 mA/g	2
CuHCF	20mM $ZnSO_4$	~95Wh/kg at 60 mA/g	~55 mAh/g<br>96.3% capacity retained after 100 cycles at 60 mA/g	9
$Zn_3[Fe(CN)_6]_2$	1M $ZnSO_4$	100 Wh/kg at 60 mA/g	~65 mAh/g<br>76% capacity retained after 100 cycles at 60 mA/g	10

α-MnO_2	1M $ZnSO_4$	--	195 mAh/g<br>70% capacity retained after 30 cycles at 10 mA/g	11
α-MnO_2	1M $ZnSO_4$	~315 Wh/kg at 32 mA/g	100 mAh/g<br>100% capacity retained after 100 cycles at 380 mA/g	12
δ-MnO_2	1M $ZnSO_4$	--	252 mAh/g<br>44% capacity retained after 100 cycles at 83 mA/g	13
α-MnO_2	1M $ZnSO_4$	--	225 mAh/g<br>63% capacity retained after 50 cycles at 83 mA/g	14
VS_2	1M $ZnSO_4$	123 Wh/kg at 50 mA/g	190.3 mAh/g<br>98% capacity retained after 200 cycles at 50 mA/g	15
$Na_3MnTi(PO_4)_3$	1M Na_2SO_4	~82 Wh/kg at 58.7 mA/g	58.4 mAh/g<br>98% capacity retained after 100 cycles at 58.7 mA/g	16
γ -MnO_2 with TiB_2	1M ZnSO4<br>saturated LiOH	--	220 mAh/g<br>55% capacity retained after 40 cycles at 0.5 mA/cm^2	17
γ -MnO_2 with TiS_2	1M ZnSO4<br>saturated LiOH	--	148 mAh/g<br>50% capacity retained after 40 cycles at 0.5 mA/cm^2	18
Manganese-based complex.	1M $ZnSO_4$<br>0.1M $MnSO_4$	335 Wh/kg at 100 mA/g<br>175 Wh/kg at 4000 mA/g	149 mAh/g<br>100% capacity retained after 500 cycles at 2000 mA/g<br>131 mAh/g<br>93.9% capacity retained after 2000 cycles at 4000 mA/g	This work

References

1. S. Guo, H. Yu, Z. Jian, P. Liu, Y. Zhu, X. Guo, M. Chen, M. Ishida and H. Zhou, *ChemSusChem*, 2014, **7**, 2115-2119.
2. N. Zhang, F. Cheng, Y. Liu, Q. Zhao, K. Lei, C. Chen, X. Liu and J. Chen, *J. Am. Chem. Soc.*, 2016, **138**, 12894-12901.
3. L. Suo, O. Borodin, T. Gao, M. Olguin, J. Ho, X. Fan, C. Luo, C. Wang and K. Xu, *Science*, 2015, **350**, 938-943.
4. W. Tang, Y. Hou, F. Wang, L. Liu, Y. Wu and K. Zhu, *Nano Lett.*, 2013, **13**, 2036-2040.
5. Q. Qu, L. Fu, X. Zhan, D. Samuelis, J. Maier, L. Li, S. Tian, Z. Li and Y. Wu, *Energy & Environmental Science*, 2011, **4**, 3985-3990.
6. Z. Hou, X. Li, J. Liang, Y. Zhu and Y. Qian, *Journal of Materials Chemistry A*, 2015, **3**, 1400-1404.

7. B. Zhang, Y. Liu, X. Wu, Y. Yang, Z. Chang, Z. Wen and Y. Wu, *Chem. Commun. (Cambridge, U. K.)*, 2014, **50**, 1209-1211.
8. V. Mathew, S. Kim, J. Kang, J. Gim, J. Song, J. P. Baboo, W. Park, D. Ahn, J. Han, L. Gu, Y. Wang, Y.-S. Hu, Y.-K. Sun and J. Kim, *NPG Asia Mater*, 2014, **6**, e138.
9. R. Trocoli and F. La Mantia, *ChemSusChem*, 2015, **8**, 481-485.
10. L. Y. Zhang, L. Chen, X. F. Zhou and Z. P. Liu, *Advanced Energy Materials*, 2015, **5**, 1400930.
11. B. Lee, H. R. Lee, H. Kim, K. Y. Chung, B. W. Cho and S. H. Oh, *Chem. Commun. (Cambridge, U. K.)*, 2015, **51**, 9265-9268.
12. C. Xu, B. Li, H. Du and F. Kang, *Angew. Chem., Int. Ed.*, 2012, **51**, 933-935.
13. M. H. Alfaruqi, J. Gim, S. Kim, J. Song, D. T. Pham, J. Jo, Z. Xiu, V. Mathew and J. Kim, *Electrochem. Commun.*, 2015, **60**, 121-125.
14. M. H. Alfaruqi, J. Gim, S. Kim, J. Song, J. Jo, S. Kim, V. Mathew and J. Kim, *J. Power Sources*, 2015, **288**, 320-327.
15. P. He, M. Yan, G. Zhang, R. Sun, L. Chen, Q. An and L. Mai, *Advanced Energy Materials*, 2017, 1601920.
16. H. Gao and J. B. Goodenough, *Angew. Chem., Int. Ed.*, 2016, **55**, 12768-12772.
17. M. Minakshi, D. R. G. Mitchell and K. Prince, *Solid State Ionics*, 2008, **179**, 355-361.
18. M. Minakshi, P. Singh, D. R. G. Mitchell, T. B. Issa and K. Prince, *Electrochim. Acta*, 2007, **52**, 7007-7013.
